# Hemin-incorporating DNA nanozyme enabling catalytic oxygenation and GSH depletion for enhanced photodynamic therapy and synergistic tumor ferroptosis

**DOI:** 10.1186/s12951-022-01617-0

**Published:** 2022-09-15

**Authors:** Xiaoxiong Xiao, Min Chen, Yuchen Zhang, Liang Li, Ying Peng, Wenhu Zhou, Junyu Li

**Affiliations:** 1 Department of Radiation Oncology, Jiangxi Cancer Hospital, Nanchang, Jiangxi China; 2 Department of Thoracic Surgery, Xiangya Hospital, Central South University, Changsha, Hunan China; 3 Xiangya Lung Cancer Center, Xiangya Hospital, Central South University, Changsha, Hunan China; 4 National Clinical Research Center for Geriatric Disorders, Changsha, China; 5 Xiangya School of Pharmaceutical Sciences, Central South University, Changsha, Hunan China; 6 Department of Thoracic Surgery, The Second People’s Hospital of Huaihua City, Huaihua, China; 7 Department of Pharmacy, Yichun People’s Hospital, Yichun, Jiangxi China

**Keywords:** Nanomedicine, DNAzyme, Aptamer, Targeting, Phototherapy, Tumor hypoxia, Catalysis

## Abstract

Photodynamic therapy (PDT) has emerged as a promising tumor treatment method via light-triggered generation of reactive oxygen species (ROS) to kill tumor cells. However, the efficacy of PDT is usually restricted by several biological limitations, including hypoxia, excess glutathione (GSH) neutralization, as well as tumor resistance. To tackle these issues, herein we developed a new kind of DNA nanozyme to realize enhanced PDT and synergistic tumor ferroptosis. The DNA nanozyme was constructed via rolling circle amplification, which contained repeat AS1411 G quadruplex (G4) units to form multiple G4/hemin DNAzymes with catalase-mimic activity. Both hemin, an iron-containing porphyrin cofactor, and chlorine e6 (Ce6), a photosensitizer, were facilely inserted into G4 structure with high efficiency, achieving in-situ catalytic oxygenation and photodynamic ROS production. Compared to other self-oxygen-supplying tools, such DNA nanozyme is advantageous for high biological stability and compatibility. Moreover, the nanostructure could achieve tumor cells targeting internalization and intranuclear transport of Ce6 by virtue of specific nucleolin binding of AS1411. The nanozyme could catalyze the decomposition of intracellular H_2_O_2_ into oxygen for hypoxia relief as evidenced by the suppression of hypoxia-inducible factor-1α (HIF-1α), and moreover, GSH depletion and cell ferroptosis were also achieved for synergistic tumor therapy. Upon intravenous injection, the nanostructure could effectively accumulate into tumor, and impose multi-modal tumor therapy with excellent biocompatibility. Therefore, by integrating the capabilities of O_2_ generation and GSH depletion, such DNA nanozyme is a promising nanoplatform for tumor PDT/ferroptosis combination therapy.

**Supplementary Information:**

The online version contains supplementary material available at 10.1186/s12951-022-01617-0.

## Introduction

The development of effective strategies to manage malignant tumors is a long-lasting medical and pharmaceutical task, given the serious threaten of this disease towards human health [1]. Currently, tumor management highly depends on surgical resection, chemotherapy, radiotherapy, or their combinations, while the prognosis of these methods is usually poor with severe side-effects, achieving only limited clinical benefits for most of the patients. To this end, various novel therapeutic modalities have extensively explored to improve the therapeutic index, and several of them have made real clinic impact. Among these promising therapeutic strategies, photodynamic therapy (PDT) has attracted particular attention owing to its advantages of excellent spatial–temporal controllability, low toxicity, non-invasiveness, and so on [2, 3]. During PDT process, the photosensitizers (PSs) are activated by light to convert the substrate of molecular oxygen into cytotoxic reactive oxygen species (ROS, mainly singlet oxygen ^1^O_2_), which causes oxidative damage towards the bio-molecules of DNA, proteins and lipid within the illuminated area [4]. The PDT-based ROS damage could eradicate tumor through various mechanisms, such as directly killing tumor cells via inducing apoptosis or necrosis, impairing the tumor vasculatures, as well as causing tumor cells immunogenic death to trigger an anti-tumor immune response [5]. Owing to these advantages, PDT has been successfully translated into clinic to treat several types of tumors [6].

However, the wide clinical applications of PDT are still limited, and its efficacy is far from satisfactory due to the complex microenvironment of solid tumors. One typical pathological feature of tumor is hypoxia [7], which completely mismatches the basic requirement of PDT. Under hypoxic condition, the photodynamic efficiency is low because of the lack of molecular oxygen substrate. Even with ROS generation, the hypoxic cells are reported to be ~ threefold more resistant to ROS damage than aerobic cells [8]. The tumor cells are also equipped with various anti-oxidant defense systems to protect them from oxidative damage, in which the commonest mechanism is to employ tumor cell abundant glutathione (GSH) to scavenge ROS [9]. Moreover, the PDT process could further aggravate the tumor hypoxia by oxygen consumption and vascular impairment, which in turn activates multiple cell survival pathways to resist PDT [10, 11]. To this end, extensive research efforts have been made to reinforce the efficacy of PDT by relieving tumor hypoxia [12, 13]. For example, several studies tried to exploit hemoglobin and perfluorocarbon nanoparticles as O_2_ carriers for tumor targeting O_2_ delivery [14, 15], while the efficiency of such transient O_2_ delivery methods is not high. Alternatively, particular research attention has been paid on the design of self-oxygen-supplying systems by using tumor abundant hydrogen peroxide (H_2_O_2_) as substrate to generate O_2_. Such catalytic oxygenation can be achieved by catalase and some catalase-mimic nanozymes [16–18], which possesses the advantages of high efficiency, tumor specificity, and constant oxygen generation. In addition, several metal-based nanozymes hold the additional benefit of GSH depletion to further enhance PDT [19, 20].

While the catalytic oxygenation shows great promise to address the key limitations of PDT, the field is still in its infancy, and there also encompasses significant problems. For instance, tedious preparation procedures are required to load and deliver catalase, and the rapid deactivation and degradation of the enzyme is still an intractable problem. Nanozymes, by contrast, are much more stable and cost-effective, and can achieved tumor targeting delivery by rational surface modifications [21, 22], while the potential toxicities such as metal poisoning strongly restrict their in vivo applications [23, 24]. Moreover, it should also consider the effective PS loading to realize co-delivery, thus making the systems even more complicated. Therefore, the development of simple yet robust self-oxygenation methods that can facilely incorporate PSs are still deemed necessary for enhanced PDT.

It has been long though that all enzymes are proteins. With the progress of nucleic acids biology, the scope of enzymes has been significantly broadened since the discovery of various nucleic acids-based enzymes, including ribozymes and DNAzymes [25–27]. Specifically, ribozymes are found in nature, while DNAzymes are artificially isolated through a combinatorial process called in vitro selection. Currently, kinds of DNAzymes that can catalyze different types of chemical reactions have been discovered [28]. Compared to protein enzymes, DNAzymes are compared favorably for in vivo applications owing to high chemical stability, reversible catalytic activity under harsh conditions (e.g., temperature, organic solvents, wide ranges of pHs), and ease of versatile modifications and labeling with low immunogenicity [26]. Moreover, such DNA-based biomaterials are highly biocompatible and programmable to assemble into DNA nanostructures with controlled size, shape and morphology based on the Watson–Crick base-pairing rules [29], which innovates a new field of DNAzyme-based catalytic therapy [30–34]. Specifically, the catalase-mimic DNAzyme has been reported, which is formed by incorporating hemin into G-rich DNA sequences, and the resulting G-quadruplex (G4)-hemin DNAzyme could effectively decompose H_2_O_2_ to generate O_2_.[35] Importantly, G4 DNA could also load chlorine e6 (Ce6, a widely used PS) with high efficiency [36], thus providing an “all-in-one” platform for in-situ oxygenation and photodynamic conversion. Strikingly, such elegant self-oxygen-supply and PS delivery system has been rarely applied for enhanced tumor PDT. The only example was reported by Yang and co-workers, who developed metal coordination-based nanoscale polymers to delivery G4 DNA systems for enhanced PDT therapy [36]. While the nanostructure is simply prepared, the coordination-based assembly system is prone to dissociation by various biological matrixes such as inorganic phosphate, metal ions, and serum [37, 38], resulting in pre-mature disassembly of the nanoparticles with inefficient tumor delivery. Therefore, it is still highly desired to develop effective delivery system for G4/hemin DNAzyme with Ce6 co-loading to allow stable in vivo circulation and tumor targeting accumulation.

**Scheme 1:**
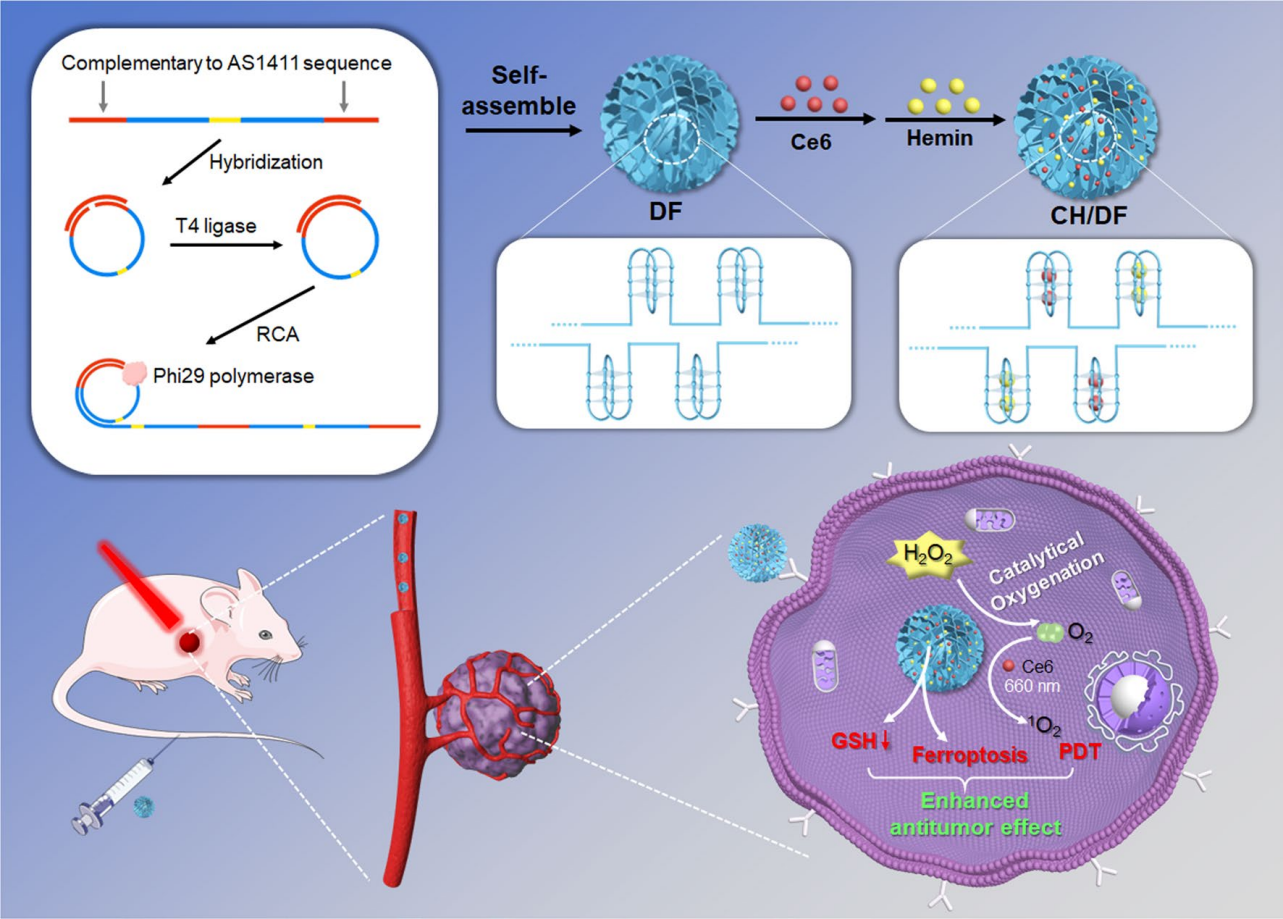
Schematical showing the preparation of DF with AS1411 G4 motif for Ce6 loading and hemin incorporation for tumor targeting PDT/ferroptosis combinatorial therapy

Recently, we and other groups have demonstrated that DNA nanoflower (DF) is a type of highly robust nanocarrier to deliver functional nucleic acids [39, 40]. DF is a spherical porous DNA nanostructure formed by rolling circle amplification (RCA). Through rational design of DNA templates and primers, arbitrary DNA sequences can be facilely encoded into DF. By virtue of its high biocompatibility, programmability and predictability, such DNA nanostructure has been explored to deliver RNA-cleaving DNAzymes for gene silencing applications [41], while its attempt on G4/hemin DNAzyme system for tumor therapy has not been reported yet. Herein, we designed and fabricated a DF with incorporation of AS1411 sequence to realize enhanced PDT (Scheme 1). AS1411 is nucleolin-binding aptamer with G-rich sequence, which has been widely used as an active ligand for tumor targeting delivery [42, 43]. AS1411 could form a typical G4 structure in DF, which was not only a cargo for hemin and Ce6 loading, but also enabled active targeting delivery of the nanosystem into tumor cells. With hemin incorporation, the DF transformed into DNA nanozyme with catalase-mimic activity for in-situ oxygenation to relief tumor hypoxia. Therefore, the AS1411 unit in DF played multiple roles of a tumor targeting aptamer, a DNAzyme motif, as well as a drug loading domain. Compared with the free G4/hemin DNAzyme, the nanozyme displayed significantly improved biological stability, which was particularly important for in vivo applications. Moreover, hemin was found to have extra benefits to deplete intracellular GSH level and induce cell ferroptosis, both of which could further synergize with PDT for even better efficacy. Upon intravenous injection, the DNA nanozyme could effectively accumulate into tumor, and impose a robust PDT/ferroptosis combinatorial therapy to inhibit tumor growth with full biocompatibility.

## Materials and methods

### Materials, cells and animals

All DNA sequences (the primer: 5ʹ-GTGGTGGTGTTGGTGGTGGT-3ʹ. the template: Phosphate-CCACCAACACCACCACCACCTTTGACACACTAGCGATACGCGTATCGCTATGGCATATCGTACGATATGCCAGTGTGTCTTTCCACCA), deoxy-ribonucleoside triphosphate (dNTP) and bovine serum albumin (BSA) were purchased from Sangon Biotech Co., Ltd (Shanghai, China). Phi29 DNA polymerase was from Lucigen Co., Ltd (USA). T4 ligase was obtained from Huamaike Bio Co., Ltd (Beijing, China). Hemin and Chlorin e6 (Ce6) were purchased from Frontier Scientific Co., Ltd (Utah, USA). Tris, KCl, NaCl, ammonium molybdate and H_2_O_2_ (30%) were from Sinopharm Co., Ltd (Shanghai, China). Singlet oxygen sensor green reagent (SOSG), 2′,7′-Dichlorofluorescin (DCFH-DA), GSH Assay Kit and Calcein-AM/PI were obtained from Solarbio Co., Ltd (Beijing, China). C11 BODIPY 581/591 was purchased from Glpbio Co., Ltd (CA, USA). Dulbecco's modified Eagle's medium (DMEM) and fetal bovine serum (FBS) were from Gibco Co., Ltd. Penicillin–streptomycin solution, 0.25% (w/v) trypsin solution, methyl thiazolyl tetrazolium (MTT) and 4′,6-diamidino-2-phenylindole (DAPI) were provided by Solarbio Co., Ltd (Beijing, China). Matrigel matrix was obtained from Biosciences Co., Ltd (New Jersey, US). Anti-Glutathione Peroxidase 4 (GPX4) Rabbit polyclonal was from Servicebio Co., Ltd (WuHan, China).

A549 cells and HEK-293 cells (human embryo kidney cells) were obtained from Xiangya cell center (Changsha, China). These cells were cultured in DMEM medium supplemented with FBS (10%), penicillin (1%, 50 U/mL) and streptomycin (1%, 50 U/mL) in a 5% CO_2_ atmosphere (37 °C).

Female Balb/c mice (6 weeks old, ≈ 20 g) were purchased from Cavans Laboratory Animal Co., Ltd (Changzhou, China) and maintained in a sterile environment and allowed free access to food and water. All animal experiments were approved by the Experimental Animal Ethics Committee of Xiangya School of Pharmaceutical Sciences of Central South University and were carried out in accordance with the requirements the National Act on the Use of Experimental Animals (People's Republic of China).

### Preparation of CH/DF

*Synthesis of circular template*: H_2_O (69.5 μL), T4 ligase buffer (10×, 10 μL), template (10 μM, 6 μL) and primer (10 μM, 12 μL) were gently mixed and incubated at 95 °C for 10 min, then cooled to room temperature. T4 ligase was added and incubated at 25 °C for 4 h to close the circular DNA gap.

*Preparation of DNA Flower (DF)*: Circular template (100 μL), dNTPs (10 mM, 40 μL), BSA (10×, 20 μL), phi29 polymerase (10 U/μL, 20 μL) and phi29 polymerase buffer (10×, 20 μL) were gently mixed in ice-bath, and incubated at 30 °C for 3 h and 75 °C for 10 min. The DNA flower was collected by centrifugation (20,000 rpm for 20 min) and washed twice with water, then mixed with buffer (20 mM Tris, 40 mM NaCl, 40 mM KCl, pH 7.6) in equal volume and incubated at room temperature for 1 h to form G4 structure.

*Preparation of Ce6, hemin-loaded DNA Flower (CH/DF)*: DF was mixed with appropriate Ce6, incubated at room temperature for 4 h, Ce6-loaded DNA nanoflower (C/DF) was collected by centrifuged (20,000 rpm, 20 min) and washed twice with buffer. The preparation method of CH/DF was the same as that of C/DF. The above products were stored at − 20 °C.

### Characterization of CH/DF

CH/DF was examined by the dynamic light scattering (DLS, Zetasizer Nano ZS90, Malvern Instruments, UK) to monitor the particle diameter, ζ-potential and polydispersity index. The morphological characteristics of CH/DF were evaluated using transmission electron microscopy (TEM, FEI, Oregon State, US) and scanning electron microscope (SEM, JSM-7900F, Tokyo, Japan). The encapsulation efficiency of drugs was calculated as follows: encapsulation efficiency = (weight of loaded drugs) / (weight of initially added drugs) × 100%. The encapsulation efficiencies of Ce6 and hemin were measured by Microplate Reader (Infinite M200, Tecan, Switzerland) and Visible–UV spectrophotometer (UV-2600, Shimadzu, Japan), respectively.

### Catalytic activity test

The catalytic activity of CH/DF was determined by the Góth method. H_2_O_2_ (0.5 mL, 1 mM) and CH/DF (0.1 mL, 0.5 μM) were mixed and reacted at room temperature for 1 min. Then ammonium molybdate solution (0.5 mL, 32.4 mM) was added to form a yellow complex. After standing for 10 min, the catalase activity of CH/DF was determined by measuring the absorbance at 350 nm.

### In vitro O_2_ production and enhanced ^1^O_2_ generation

To study the self-producing O_2_ performance of CH/DF or CH/G4, the O_2_ production was monitored the portable dissolved oxygen meter (JPBJ-609L, INESA Scientific Instrument Co., Ltd., China) every 10 s for 90 s. When O_2_ level did not change, laser irradiation (660 nm, 0.75 W/cm^2^) was added to study the dynamic change of O_2_ of CH/DF. The ^1^O_2_ production was tested by singlet oxygen sensor green (SOSG) probe after laser irradiation. Briefly, CH/DF (0.8 mL, 0.5 μM) was mixed with SOSG solution (0.1 mL, 25 μM). Then, H_2_O_2_ (0.1 mL, 100 mM) was added, and a continuous laser at 660 nm was applied with a power of 0.75 W/cm^2^ every 10 s for 50 s. The fluorescence intensity of SOSG was measured by a fluorescence spectrophotometer (Ex = 490 nm, Em = 525 nm). To study the biological stability of the DNAzyme, the CH/DF or CH/G4 were pretreated with 10% FBS for 10 h, followed by the treatments as described above.

### Cellular uptake study

A549 cells were seeded in 24-well plate at a density of 2 × 10^5^ cells per dish overnight. Subsequently, CH/DF was added to incubated for 1 h, 2 h or 4 h. After washing three times with PBS, the cells were stained with DAPI, and the fluorescence was observed by fluorescence imaging system (Model No. CYTATION5, BioTek). Moreover, A549 cells and HEK-293 cells were utilized to investigate the specific uptake of CH/DF for tumor cells. To study the cell uptake mechanism of DF, a variety of inhibitors (chlorpromazine: the clathrin inhibitor; colchicine: the macropinocytosis inhibitor; nystatin: the caveolin inhibitor; NaN_3_: ATP inhibitor) were used to intervene the endocytosis pathway. A549 cells were seeded with 2 × 10^5^ cells per well in 24-well plate and incubated overnight. The cells were treated with chlorpromazine (10 µg/mL), colchicine (5 µg/mL), nystatin (15 µg/mL) or NaN_3_ (1 mg/mL) for 30 min, and then CH/DF was added and incubated for 2 h. Fluorescence was observed and quantified by fluorescence imaging system (Model No. CYTATION5, BioTek).

### In vitro cytotoxicity study

MTT assay was used to evaluate the cytotoxic effects of CH/DF to A549 cells. A549 cells were seeded with 5 × 10^3^ cells per well in 96-well plate and incubated overnight, and then treated with a series of concentration dilutions of DF, C/DF and CH/DF (Ce6: 0.45, 0.9, 1.8, 3.6 and 7.2 μM; Hemin: 0.02, 0.04, 0.08, 0.16 and 0.32 μM) for 48 h. The C/DF and CH/DF groups were irradiated with laser (0.75 W/cm^2^, 1 min) after incubation for 24 h. After that, the cells were washed twice with PBS and treated with the MTT reagent (5 mg/mL, 10 μL) for 4 h. Subsequently, the medium was removed and dimethyl sulfoxide (DMSO, 150 μL) was added. Finally, the UV–vis absorbance of each well was measured by Microplate Reader, and the cells viability was computed using the following formula:

Cell viability = (A_sample_/A_control_) × 100%, where A represents the absorbance at 570 nm.

To study the effect of ferroptosis inhibitors or inducers t, the cells were co-treated with CH/DF (Ce6: 1.6 μM) plus ferrostatin-1 (1 μM), glutathione (GSH, 1 mM), glutamic acid (1 mM), or erastin (10 μM), respectively. After culturing for 48 h, MTT assay was performed to measure the cell viability.

### Live/Dead Staining assay

A549 cells were seeded with 2 × 10^5^ cells per well in 12-well plate and treated with different formulations. The cells without any treatment were used as control. Subsequently, the cells were stained with both Calcein AM and PI, and observed by inverted fluorescent microscope (NIKON, Ti-S, Japan).

### Intracellular ROS and LPO generation

DCFH-DA was used to evaluate the generation of intracellular ROS. A549 cells were seeded with 2 × 10^5^ cells per well in 24-well plate and treated with different formulations (Ce6: 5 μM) for 6 h. The cells were washed with PBS three times and incubated with DCFH-DA (10 μM) for 30 min, and then irradiated for 1 min (0.75 W/cm^2^). Finally, the fluorescence of DCFH-DA of cells was observed by fluorescence imaging system (Model No. CYTATION5, BioTek). C11 BODIPY 581/591 probe was used to detect LPO, and the detection method was similar to ROS.

### Intracellular GSH level measurement

GSH assay kit was used to quantify the intracellular GSH level. A549 cells were seeded with 2 × 10^5^ cells per well in 24-well plate and treated with different formulations (Hemin: 2 μM) for 24 h. Then, the C/DF and CH/DF groups were irradiated with laser and incubated for another 1 h. All cells were collected and lysed by liquid nitrogen, and the supernatants were collected by centrifugation (12,000 rpm, 10 min). Then, the supernatants were treated GSH assay kit and the absorbances were measured at 412 nm by Microplate Reader (Infinite M200, Tecan, Switzerland).

### Western blot analysis of HIF-α protein expression

A549 cells were seeded with 4 × 10^6^ cells per well in 6-well plate and treated with different formulations for 24 h. Cells were treated by RIPA buffer to collect total protein. Protein concentrations were determined by a BCA Protein Assay Kit (Dingguo changsheng, China). Then, proteins were run on polyacrylamide gel and were transferred to PVDF membranes. After blocking with 5% skim milk, the membranes were incubated with HIF-α polyclonal antibody and β-actin antibody overnight at 4 °C, and then incubated with horseradish peroxidase-conjugated secondary antibody for 1 h at room temperature. Finally, the proteins were visualized by the ChemiDoc MP Imaging System (Bio-Rad).

### Construction of A549 xenograft tumor model

Six-week-old female BALB/c nude mice were used to establish the A549 xenograft tumor model. Briefly, A549 cells were collected and dispersed in PBS at a density of 2 × 10^7^/mL, and then injected into the skin of mice subcutaneously (100 μL per mouse).

### Biodistribution analysis

To study biodistribution, free Ce6 or CH/DF (Ce6: 2 mg/kg) was injected to A549 tumor-bearing mice intravenously. After 24 h administration, the mice were sacrificed and their primary organs (heart, liver, spleen, lung and kidney) and tumors were collected for ex vivo imaging with the IVIS Lumina XRMS Series III system (PerkinElmer, Waltham, MA).

### In vivo antitumor efficacy and histological analysis

The A549 tumor-bearing mice with tumor volumes of approximately 100 mm^3^ were randomly divided into 6 groups (n = 6 per group): (1) PBS group as the control; (2) DF; (3) C/DF; (4) C/DF + L; (5) CH/DF; (6) CH/DF + L (Ce6: 2.5 mg/kg, respectively). The preparations were injected through a tail vein, and tumors were irradiated with a laser irradiation (0.75 W/cm^2^, 5 min) after 24 h of administration. Tumor sizes and body weights were recorded every other day after injection. Tumor volumes were calculated as follows: Volume = (length × width^2^)/2.

On the 16th day, all mice were sacrificed, tumors were collected for the hematoxylin and eosin (H&E) staining, TdT-mediated dUTP nick-end labeling (TUNEL) staining, immunofluorescence (caspase-3 and GPX4) staining. All major organs were collected for H&E staining to evaluate the safety of formulations.

### Statistical analysis

The data were expressed as mean ± SD on the basis of at least three independent experiments. One-way ANOVA analysis of variance was used to determine the statistical significance of the difference group. P value < 0.05 was considered statistically significant.

## Results and discussions

### Preparation and characterizations of CH/DF

**Fig. 1:**
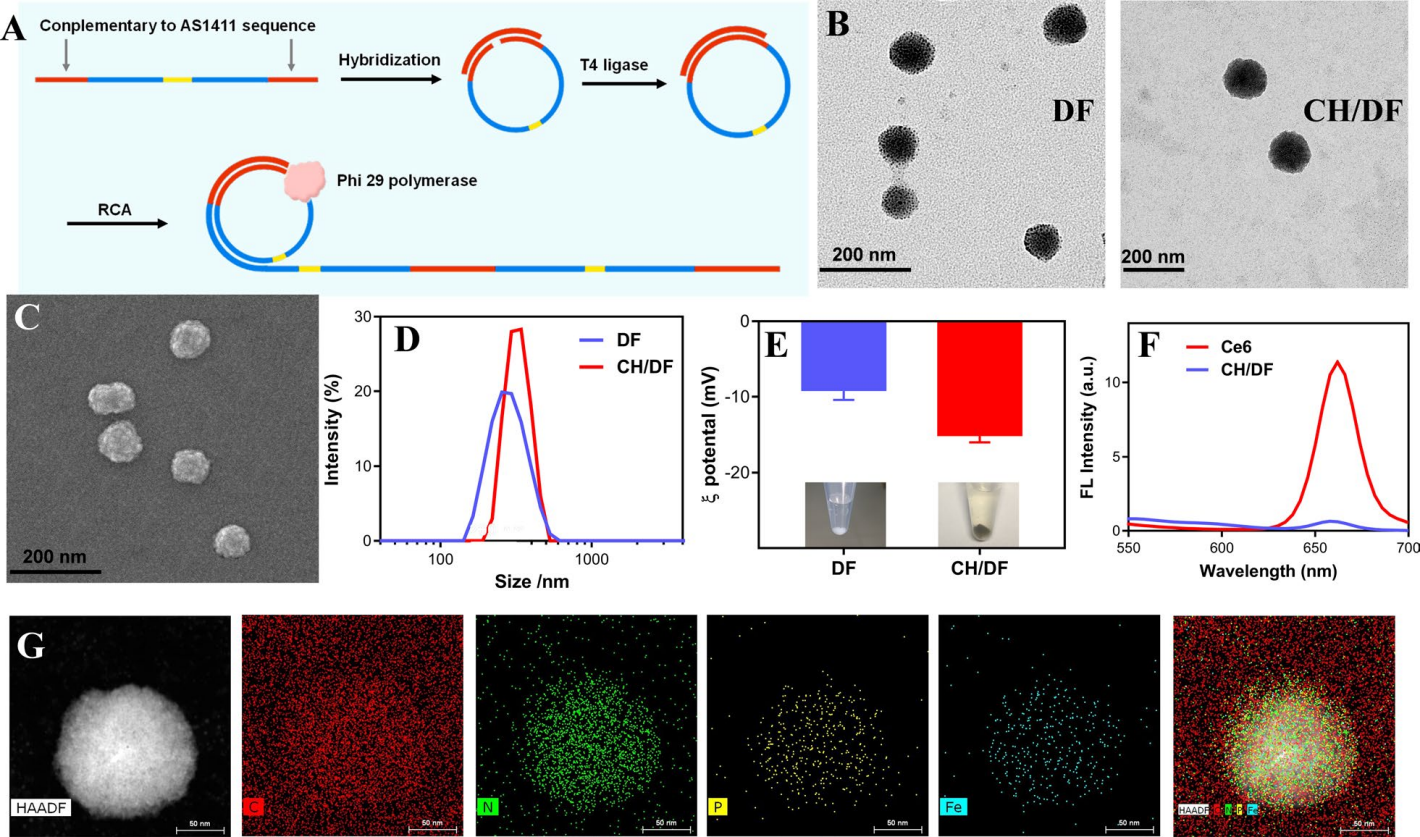
**A** Schematic showing the design of template sequence and the preparation of DF. **B** The TEM micro-images of DF and CH/DF. **C** The SEM micro-image of CH/DF. **D** DLS and **E** surface charge measurements of DF and CH/DF. Inset: the appearance of the nanoparticles after centrifugation. **F** The fluorescence spectra of Ce6 before and after loading into DF. **G** The elemental mapping micro-image of CH/DF

The DF were prepared according to our previous report [39], in which the circular DNA template was first ligation by T4 ligase, followed by rolling circle amplifications (Fig. 1A). With rational sequence design of the template, the resulting DF contained a AS1411 aptamer region for functionalization [42, 43], and a double helix region for structure stabilization. The successful preparation of DF was dynamically monitored by hydrodynamic size, and TEM, gel electrophoresis (Additional file 1: Figure S1). The nanoparticles were rapidly formed in 3 h, while the size gradually increased from 100 to 350 nm based on TEM characterization. For effective tumor targeting delivery via EPR effect, the reaction time was chosen at 3 h with smaller particle size [44], and the structure of the resulting DF were presented in Fig. 1B and C, showing spherical morphology with uniformed size. Then, the photosensitizer of Ce6 and hemin were coloaded into DF via intercalation into AS1411 domain though π-π stacking, [36, 45] and the obtained CH/DF displayed slightly increased particle size (Fig. 1D), and even more negative surface charge (Fig. 1E). From TEM, the morphology and high dispersity of nanoparticles were maintained after Ce6 and hemin loading (Fig. 1B), while the appearance was changed from white color to purple (inset in Fig. 1E, the precipitants), suggesting the Ce6/hemin loading. Over a period of 24 h incubation, no obvious size change was observed in both PBS buffer and FBS-containing medium (Additional file 1: Figure S2), confirming its high colloidal stability for biological applications.

Upon loading into DF, the fluorescence intensity of Ce6 significantly weakened (Fig. 1F), attributable to the fluorescence quenching effect of G-quadruplex (G4) structure. Each DNA nanoflower was calculated to load 200 Ce6 molecules. In addition, the drug release profile of Ce6 was studied. Notably, Ce6 showed a typical pH-responsive release profile (Additional file 1: Figure S3), in which only 10% of the drug was released at pH 7.4 after 12 h, while the release significantly accelerated at pH 5.5. Such property is advantageous for prolonged in vivo circulation and rapid drug release after being delivered at target site. Meanwhile, the successful loading of hemin can be confirmed by the elemental mapping, in which the Fe and P signal was originated from hemin and the DNA payload, respectively (Fig. 1G).

### In-situ oxygenation by DNA nanozymes for enhanced PDT

**Fig. 2:**
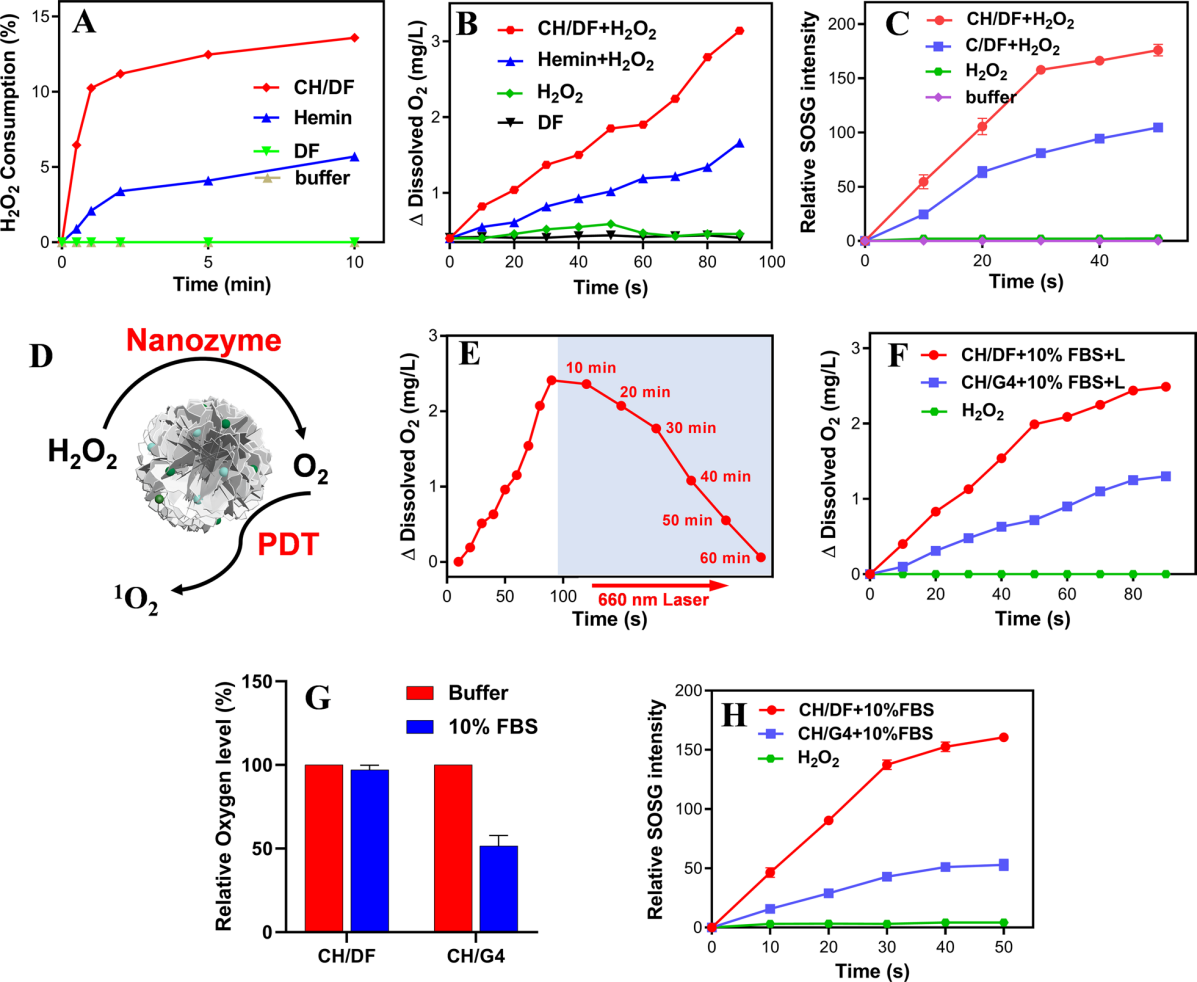
**A** H_2_O_2_ consumption kinetics in presence of CH/DF, DF, and the buffer control. The kinetics of **B** O_2_ and **C** ^1^O_2_ generation for different reaction groups. **D** A carton showing the catalytic oxygenation for enhanced PDT. **E** The conceptual demonstration of self-oxygen-supply system. H_2_O_2_ was catalytically decomposed into molecular oxygen, and the laser was performed at 10 min for constant 60 min. **F** Catalytic oxygen generation for CH/DF and CH/G4 with 10% FBS pretreatment. **G** Relative amount of oxygen generation catalyzed by CH/DF and CH/G4 for 90 s with or without pretreatment of 10% FBS. **H** ^1^O_2_ generation upon laser irradiation for CH/DF and CH/G4 with 10%FBS pretreatment

Upon intercalation of hemin into AS1411, a typical G4/hemin DNAzyme can be formed with peroxidase like activity [46]. Therefore, the CH/DF contained multiple DNAzyme units, which can be regarded as a type of DNA nanozymes. Such nanozyme could rapidly decompose H_2_O_2_ (Fig. 2A), accompanied by the generation of molecular oxygen (Fig. 2B). Without hemin loading, by contrast, the control DF did not show any catalytical activity, further confirming the formation of G_4_/hemin DNAzyme. Free hemin also showed peroxidase characteristic, which is consistent with previous report [47]. While they share the same basic mechanism through oxidation–reduction of iron in hemin structure upon reaction with H_2_O_2_, the activity was strongly enhanced upon incorporation into G4 structure to form DNAzyme. We further tested the multiple turn-over of the reaction, and the nanoparticles could achieve similar O_2_ generation rate after 3 cycles (Additional file 1: Figure S4), confirming its catalytic activity. The in-situ generated oxygen could in turn provide oxygen substrate for PDT. To demonstrate this, the PDT efficacy was monitored by measuring single oxygen (^1^O_2_) production using a SOSG probe. Upon laser irradiation, both C/DF (the DF with Ce6 alone loading) and CH/DF showed considerable efficacy for ^1^O_2_ production (Additional file 1: Figure S5), indicating the photodynamic activity of Ce6 was retained after encapsulation into DF, although its fluorescence was quenched. Notably, further addition of H_2_O_2_ could significantly accelerate the efficacy of CH/DF but leaving C/DF being unaffected (Fig. 2C), which can be explained by DNAzyme-mediated catalytic generation of oxygen for enhanced PDT. Note that the tumor microenvironment was abundant with H_2_O_2_ owing to the Warburg effect of tumor cells [48]. Therefore, such enzyme system could act as an in-situ oxygen supplier at tumor site by employing tumor abundant H_2_O_2_ for effective PDT (Fig. 2D). To directly verify such oxygen-self-supply system, the H_2_O_2_ was added to allow O_2_ generation, followed by a constant laser irradiation for 60 min (Fig. 2E). The O_2_ level was still higher than the baseline in the end, demonstrating that the O_2_ generation was sufficient for long-time PDT.

While DNA-based biomaterials are highly biocompatible, their in vivo applications are restricted due to non-specific interactions and degradation. For example, we previous found that serum proteins could bind with DNAzyme and affect its activity [49]. Upon integrating into DF, biological stability of the DNAzyme could be significantly enhanced, which can be attributed to the dense packaging of DNA in nanoflowers to prevent direct contact between nuclease and the inner layer of DNA molecules and retard the digestion process to a great extent [50, 51]. To verify this, we compared the catalytic activity of free G4/hemin DNAzyme and the DNAzyme-embedded DF. For parallel comparison with CH/DF, the same concentration of G4 DNA was used with equal loading amount of both Ce6 and hemin (termed CH/G4). As such, their catalytic activity and PDT effect were comparable in solution (Additional file 1: Figure S6). We then challenged them with serum to mimic in vivo circulation. After 12 h incubation, the catalytic activity of CH/DF was still maintained, while free CH/G4 DNAzyme displayed an obvious decrease of kinetics (Fig. 2F), indicating the deactivation of DNAzyme by biological matrixes. We previously showed that DNAzyme has high chemical stability in serum, but strong protein binding could passivate the enzymatic activity [49], which may explain the decrease of CH/G4 activity in serum. As a result, the oxygen generation was markedly reduced by twofold (Fig. 2G). Such different can be further reflected by ^1^O_2_ generation upon laser irradiation, in which CH/DF exhibited much better PDT efficacy than CH/G4 after FBS pre-treatment (Fig. 2H). Therefore, DF could protect DNAzyme from deactivation under biological matrixes, and provides a robust nanocarriers to facilely integrate functional nucleic acids for in vivo applications.

### Aptamer-mediated tumor cells targeting delivery

**Fig. 3:**
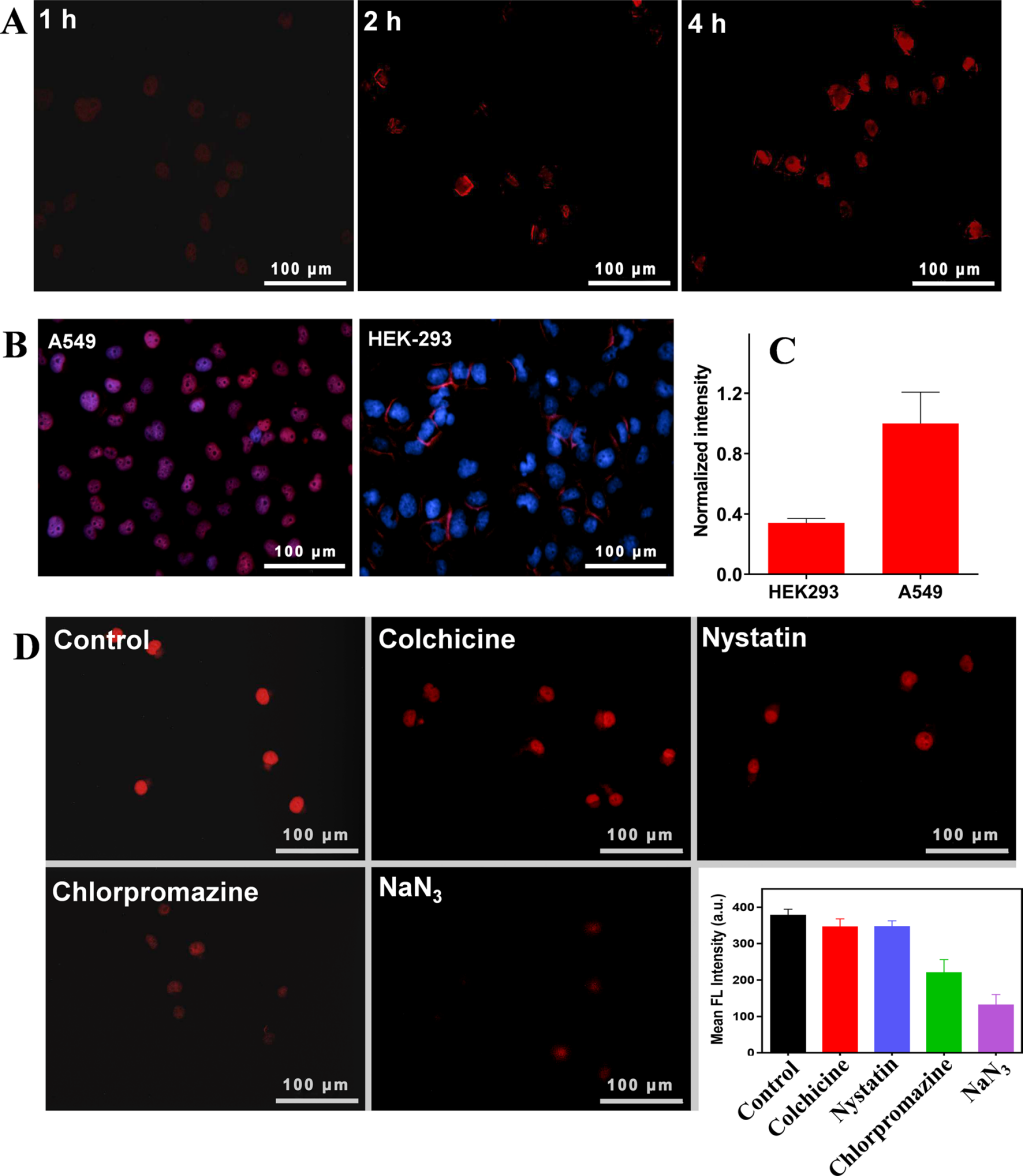
**A** Fluorescence images showing the time-dependent internalization of the CH/DF into A549 cells. **B** Internalization of the nanoparticles by A549 and HEK-293 cells and **C** the intensity quantification. **D** Fluorescence images and intensity quantification indicating the effect of various probe ligands on cellular internalization of the nanoparticles

After systematical characterizations at test tube level, we then explored the intracellular performances of the nanoparticles. Each DF contained multiple AS1411 sequences in its structure, which was not only a domain for payloads loading, but also an active ligand for tumor targeting delivery by virtue of its high affinity with nucleolin that are overexpressed on tumor cells surface [52]. As a proof-of-concept demonstration, the nucleolin overexpressed A549 cancer cells were chosen. The intracellular transportation of the nanoparticles can be conveniently visualized owing to the intrinsic fluorescence of the loading Ce6. The fluorescence was gradually intensified overtime (Fig. 3A), and a bright signal was observed after 4 h incubation, indicating the effective nanoparticles internalization. We then tested the tumor targetability, and the HEK-293 normal cells were used as control. After 4 h incubation, obviously brighter fluorescence was observed in tumor cells than normal cells (Fig. 3B), resulting in ~ threefold higher intensity (Fig. 3C). Interestingly, we noticed a high co-localization of the nanoparticles with the cell nuclei, indicating the effective intranuclear delivery. This can be attributable to high binding affinity of AS1411 for nucleolin to enable the subsequent intranuclear delivery. Since oxidative destruction of DNA is the important mechanism of PDT to kill tumor cells, such intranuclear delivery would enable the ROS generation within cell nuclei to directly destroy DNA for better PDT efficacy. To have a fundamental understanding, the delivery pathway was further explored by using various cell delivery pathway inhibitors (Fig. 3D) [53, 54]. Pre-treatment of colchicine and nystatin has little effect on internalization of the nanoparticles, indicating the minimal contribution of microtubular- and caveolae-mediated endocytosis [54]. With chlorpromazine pretreatment, by contrast, the intracellular fluorescence was markedly weakened, consistent with previous report that DF was mainly delivered via clathrin-mediated endocytosis [41]. In addition, such pathway was energy-dependent as evidenced by the strong influence of NaN_3_ pre-treatment.

### Synergistic anti-tumor effect via PDT/ferroptosis combinatorial therapy

**Fig. 4:**
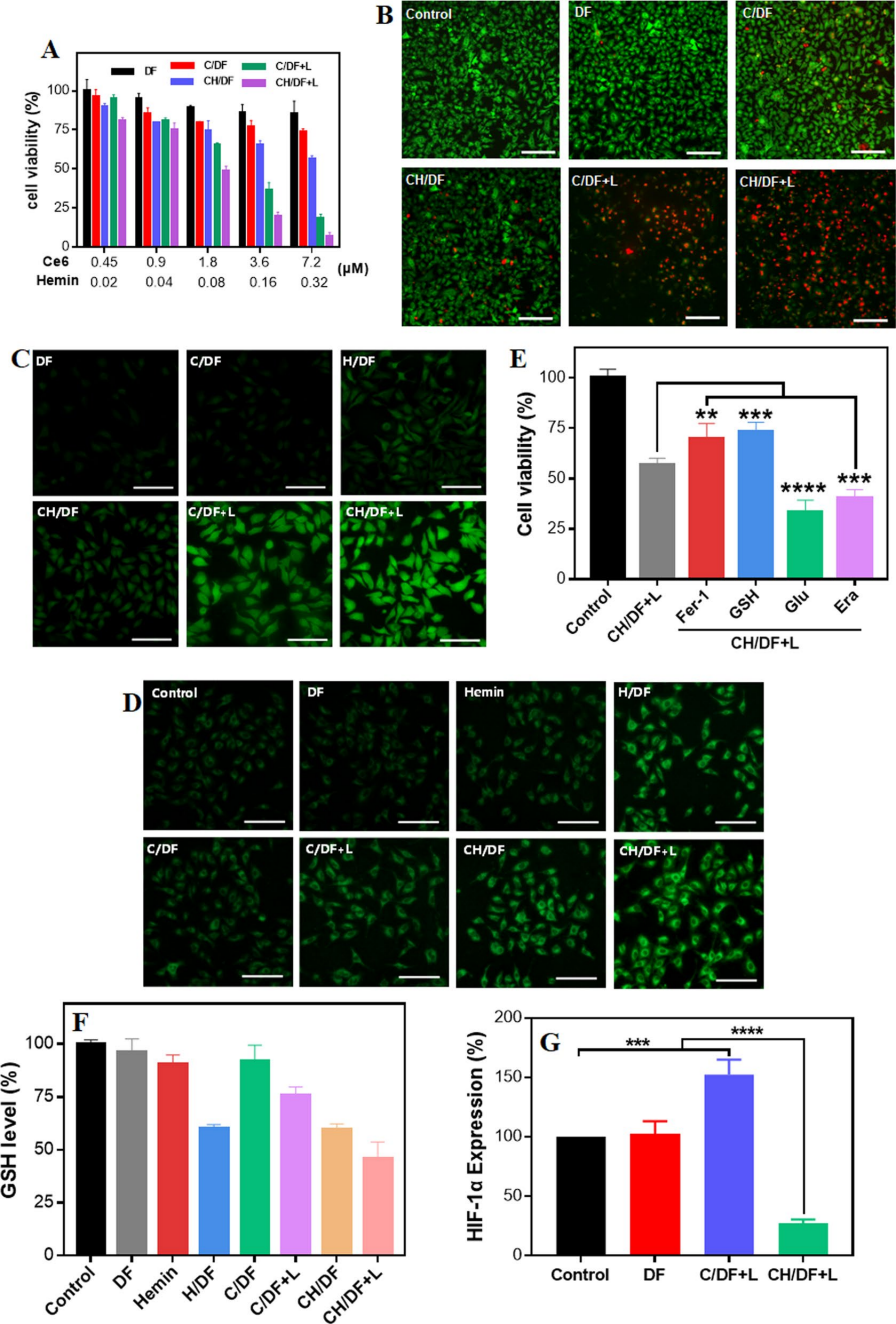
**A** The cytotoxicity of various treatments towards A549 tumor cells. **B** Calcein AM/PI double stain of A549 cells after various treatments. Scale bar, 200 μm. **C** Fluorescence images of the cells stained by DCFDA to probe ROS generation after various treatments. Scale bar, 100 μm. **D** Fluorescence images of the cells stained by BODIPY-C11 to probe LPO accumulation after various treatments. Scale bar, 100 μm. **E** The effect of various ferroptosis inhibitors/inducers on cytotoxic activity of CH/DF (with laser) towards tumor cells. **G** The expression of HIF-1α protein after various treatments

Having demonstrated the tumor targeting delivery, we then investigated the anti-tumor efficacy. Based on the MTT assay, DF was highly comparable with non-cytotoxicity on cells, while C/DF elicited marginal phototoxicity during treatment (Fig. 4A). CH/DF also showed noticeable cell suppression effect, which can be ascribed to the Fe-containing hemin to induce cell ferroptosis. Upon exposure to laser, the Ce6-loading DF showed concentration-dependent anti-tumor effect. Notably, CH/DF displayed much better efficacy than C/DF with significant decrease of IC_50_ value, suggesting the synergistic effect between each therapeutic modality. To confirm such result, Calcein-AM/PI co-staining was performed to visualize live and dead cells with green and red fluorescence, respectively (Fig. 4B). Bright green fluorescence was seen in DF group, in line with the high biocompatibility of such DNA-based carriers. For C/DF and CH/DF group, sparse red fluorescence was noticed, while the signal became strongly intensified after laser irradiation, in which all these observations were highly consistent with the above MTT assay.

Next, the anti-tumor mechanisms were studied in detail. Both PDT and ferroptosis could damage cells via generating large amount of ROS to damage cells, so the ROS level was first probed by using 2′,7′-dichlorofluorescin diacetate (DCFDA) with green fluorescence signal. The background ROS level was quite low, and DF or C/DF treatments barely showed any fluorescence (Fig. 4C). Notably, the green fluorescence turned on upon treatment with H/DF or CH/DF, owing to hemin-induced ferroptosis. The fluorescence was further enhanced in combination of PDT via laser irradiation, demonstrating their co-contribution for ROS generation.

To further identify the ferroptosis-based cell death pathway, intracellular lipid peroxidation (LPO) level, the specific indicator of ferroptosis, was probed by BODIPY-C11 (Fig. 4D). Obviously, all hemin-containing DF could produce green fluorescence inside cells, indicating LPO accumulation. Meanwhile, the LPO signal was further enhanced upon laser irradiation, confirming the sensitizing effect of PDT on ferroptosis. We also noticed that H/DF could produce much stronger fluorescence than free hemin, emphasizing the advantages of nanoparticles-mediated tumor cells targeting delivery. The contribution of ferroptosis-based anti-tumor effect was further examined by adding various ferroptosis inhibitors and promoters. Specifically, cytotoxicity of the nanoparticles was passivated upon addition of both ferroptosis inhibitor of ferrostatin-1 (Fer-1) and antidote of glutathione (GSH), but was enhanced by the promoters of glutamate (Glu) and erastin (Era) (Fig. 4E) [30]. All these results demonstrated the critical contribution of hemin-induced ferroptosis for tumor therapy.

For ROS-based anti-tumor mechanism, one key limitation is the GSH-mediated cell resistance [55]. Tumor cells has high GSH concentration (~ 10 mM), which could effectively scavenge a wide range of ROS to rescue ROS-induced cell damage. To this end, various GSH exhausting strategies have attempted to reinforce the efficacy of ferroptosis/PDT [56]. Fortunately, hemin has been reported to possess intrinsic activity to deplete GSH [57], which could benefit the therapeutic efficacy. To confirm such capability, we measured the intracellular GSH level. As expected, H/DF could effectively decrease the intracellular GSH level as compared to DF control (Fig. 4F). Interestingly, free hemin has little effect on GSH level, likely due to the fact that the negatively charged hemin is repelled by cell membrane with minimal internalization. Therefore, the incorporation of hemin into DF could facilitate its intracellular delivery.

In addition, the loading of hemin into AS1411 forms of peroxidase DNAzyme for in-situ oxygenation, which could solve the other restriction of PDT, i.e., tumor hypoxia. To demonstrate this concept, HIF-1α protein, the biomarker of the tumor hypoxia, was measured (Fig. 4G). Without any treatment, the tumor cells showed a considerable HIF-1α protein expression, and the hypoxia was further exacerbated with C/DF plus laser treatment, which can be attributable to the O_2_ consumption during PDT. For CH/DF group, by contrast, the HIF-1α protein level was significantly reduced even under laser irradiation condition, confirming the capability of such self-oxygen-supply nanozyme to relief tumor hypoxia, which is highly important for tumor PDT in vivo.

### Targeting delivery of CH/DF for synergistic anti-tumor therapy in vivo

**Fig. 5:**
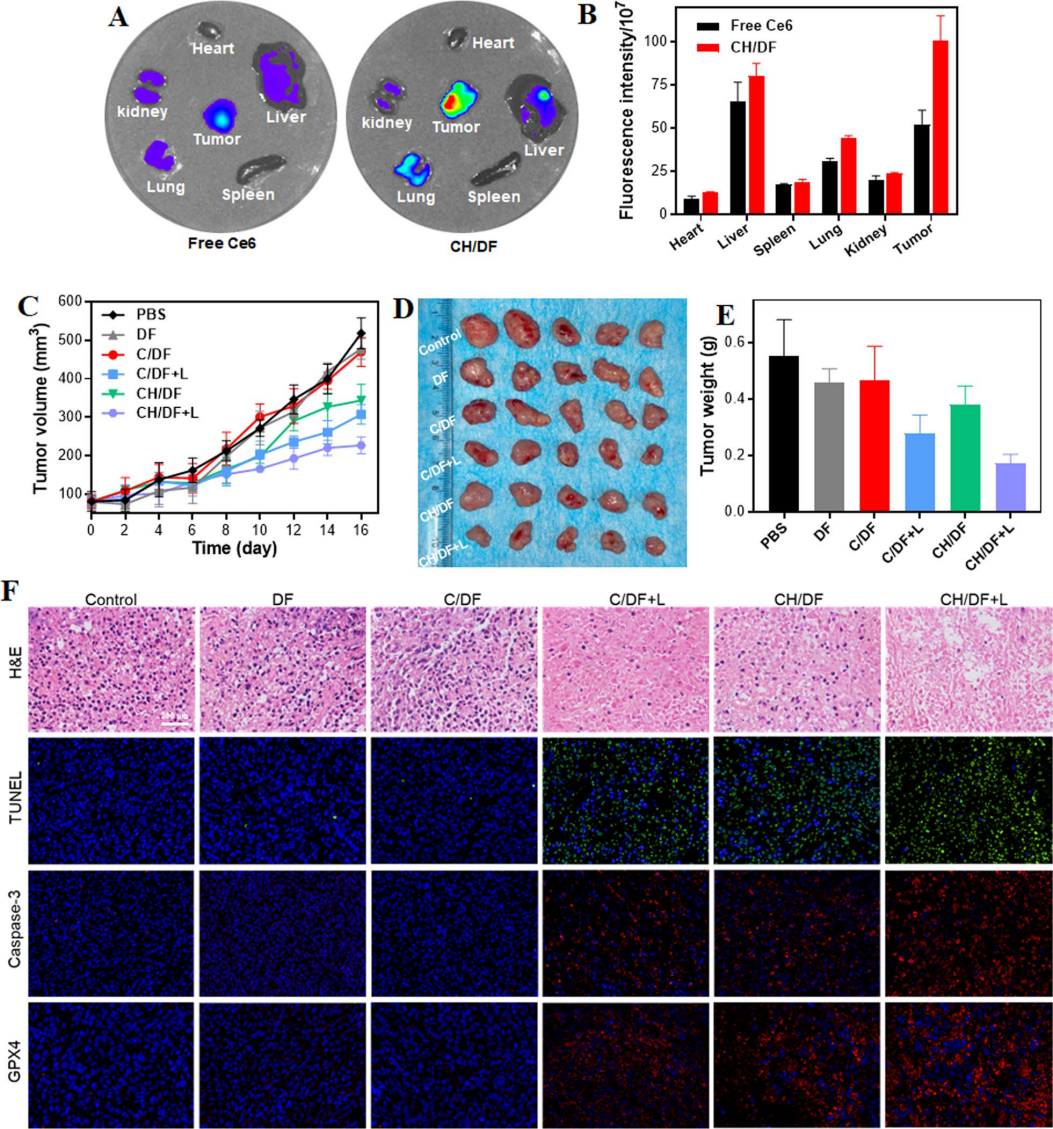
**A** Ex vivo imaging the biodistribution of CH/DF and free Ce6 at 12 h post-injection into tumor bearing mice. **B** The intensity quantification at different organs based on the fluorescence images in **A**. **C** Dynamic monitoring the tumor growth after various treatments. **D** The appearance and **E** weight of the tumors at day 16 post various treatments. **F** Tumor H&E staining images, fluorescent staining of TUNEL and caspase-3 of the mice with different treatments

We next explored the in vivo performance of the nanosystem by using nude mice bearing subcutaneous A549 tumors. The biodistribution of CH/DF after intravenous injection was studied by fluorescence imaging to track the delivery of Ce6-containing nanoparticles. Due to the light penetration issue, the images were taken by collecting the main organs as well as tumor tissue for ex vivo observation (Fig. 5A). After 24 h circulation, the liver showed the highest fluorescence intensity, since liver is the major organ to trap, metabolize, and eliminate nanoparticles [58]. Importantly, compared with free Ce6, CH/DF showed notably bright fluorescence at tumor site. We then quantified the intensity, and ~ twofold higher accumulation was observed for Ch/DF (Fig. 5B), demonstrating the tumor targeting delivery of the nanoparticles. Such targetability can be attributable to both EPR effect for passive accumulation and aptamer-mediated tumor cells selective recognition.

Motivated by the above results, we further applied the nanoparticles for tumor therapy. When the tumor volume reached ~ 100 mm^3^, the mice were randomly divided into six groups, each receiving one of the following treatments with a single dose injection: PBS control, DF, C/DF, C/DF, C/DF plus laser, CH/DF, and CH/DF plus laser. Each formulation was administrated by intravenous injection, and only one dose was given. To monitor the efficacy, the tumor tissue was dynamically measured every other day, based on which the tumor growth curve was obtained (Fig. 5C). Without drugs loading, the DF merely showed any influence on tumor growth. C/DF without laser also failed to show any efficacy, while the tumor growth was suppressed upon laser irradiation, attributable to PDT effect. For CH/DF without laser, we also observed a notable tumor suppression activity, which was originated from hemin-mediated ferroptosis. With laser irradiation, the CH/DF showed an even stronger inhibition of tumor growth was seen, suggesting a combinatorial efficacy between ferroptosis and PDT. For direct observation, the mice were sacrificed and the tumor were collected for weighting (Fig. 5D, E), in which the results were highly consistent with the in vivo observation. Among various treatments, the CH/DF plus laser achieved the best therapeutic efficacy.

Then, the therapeutic efficacy was also evaluated by hematoxylin and eosin (H&E), TUNEL and caspase-3 staining of the tumors after treatments (Fig. 5F). Compared with the control, both DF and C/DF did not induce much pathological changes, confirming the high biocompatibility of the nanosystems. For the C/DF plus laser and CH/DF groups, by contrast, the tumors showed obvious necrotic responses, accompanied by the strong TUNEL and caspase-3 fluorescence, owing to the PDT and ferroptosis effect, respectively. Among them, CH/DF plus laser achieved the highest level of necrosis, apoptosis, and caspase-3 signal, verifying the combination effect of PDT/ferroptosis for enhanced anti-tumor therapy. Finally, the toxicity of each treatment was briefly studied. The body weight of all treating mice was unchanged during therapy (Additional file 1: Figure S7), and all major organs do not observe any obvious pathological abnormalities based on the H&E staining images (Additional file 1: Figure S8), indicating the high biocompatibility of the DF-based nanosystems with minimal side-effects.

## Conclusions

In summary, we developed a DNA nanoreactor to simultaneously address several key limitations of PDT. The DNA nanostructure was facilely prepared via a well-defined protocol, and systematically characterized with uniformed size and morphology. All biological functions of such DNA nanostructure were derived from its multiple G4/hemin DNAzymes incorporation. First, as a type of catalase mimic DNAzyme, it allowed in situ self-oxygen-supply for enhanced PDT, and importantly, such nano-catalyst could resist biological degradation to enable in vivo applications. Second, the AS1411 G4 could also act as an active targeting ligand to mediate tumor cells selective internalization and intranuclear transportation for better therapeutic efficacy. Moreover, the loading hemin possessed extra functions to deplete intracellular GSH and induce cell ferroptosis, both of which synergized the anti-tumor effect of PDT. The nanostructure was further applied in tumor-bearing mice, which showed tumor targeting delivery and tumor growth inhibition via multiple anti-tumor mechanisms. Given its excellent biocompatibility and facile preparation, such DNA-based nanostructure holds great promise as multi-functional platform for tumor therapy.

## Supplementary Information

**Additional file 1.** Characterization of DF formation by size, TEM and gel electrophoresis; colloidal stability of the nanoparticles; kinetics of drug release; catalytic multiple turn-over of the nanozymes; PDT effect; body weight change during treatments; H&E staining of the major organs.

## Data Availability

The raw data and processed data required to reproduce these findings are available from the corresponding author upon request.
