# Correction: Hemin‑incorporating DNA nanozyme enabling catalytic oxygenation and GSH depletion for enhanced photodynamic therapy and synergistic tumor ferroptosis

**DOI:** 10.1186/s12951-022-01678-1

**Published:** 2022-12-02

**Authors:** Xiaoxiong Xiao, Min Chen, Yuchen Zhang, Liang Li, Ying Peng, Junyu Li, Wenhu Zhou

**Affiliations:** 1 Department of Radiation Oncology, Jiangxi Cancer Hospital, Nanchang, China; 2 Department of Thoracic Surgery, Xiangya Hospital, Central South University, Changsha, China; 3 Xiangya Lung Cancer Center, Xiangya Hospital, Central South University, Changsha, China; 4 National Clinical Research Center for Geriatric Disorders, Changsha, China; 5 Xiangya School of Pharmaceutical Sciences, Central South University, Changsha, China; 6 Department of Thoracic Surgery, The Second People’s Hospital of Huaihua City, Huaihua, China; 7 Department of Pharmacy, Yichun People’s Hospital, Yichun, China

**Correction to: *Journal of Nanobiotechnology 20,*** 1410 (2022)

10.1186/s12951-022-01617-0

Following publication of the original article [1], the author would like to add Junyu Li as co-corresponding author.

The author group has been updated above and the original article [1] has been corrected.
