# Supplementary material for: Hemin-incorporating DNA nanozyme enabling catalytic oxygenation and GSH depletion for enhanced photodynamic therapy and synergistic tumor ferroptosis
Source: J Nanobiotechnology. 2022 Sep 15;20:410. doi: 10.1186/s12951-022-01617-0 (PMC9479271; doi:10.1186/s12951-022-01617-0)
Supplement: Supplementary file 1 — Additional file 1. Characterization of DF formation by size, TEM and gel electrophoresis; colloidal stability of the nanoparticles; kinetics of drug release; catalytic multiple turn-over of the nanozymes; PDT effect; body weight change during treatments; H&E staining of the major organs. [file 12951_2022_1617_MOESM1_ESM.docx]

**Supporting Information**

**Hemin-incorporating DNA Nanozyme Enabling Catalytic Oxygenation and GSH Depletion for Enhanced Photodynamic Therapy and Synergistic Tumor Ferroptosis**

Xiaoxiong Xiao^2, 3, 4^, Min Chen^6^, Yuchen Zhang^7^, Liang Li^5^, Ying Peng^5^, Junyu Li^1^*, Wenhu Zhou^3, 5^*

^1^. Department of Radiation oncology, Jiangxi Cancer hospital, Nanchang, Jiangxi Province, China

^2^. Department of Thoracic Surgery, Xiangya Hospital, Central South University, Changsha, Hunan, China

^3^. Xiangya Lung Cancer Center, Xiangya Hospital, Central South University, Changsha, Hunan, China

^4^. National Clinical Research Center for Geriatric Disorders, Changsha, China

^5^. Xiangya School of Pharmaceutical Sciences, Central South University, Changsha, Hunan, China

^6^. Department of Thoracic Surgery, The Second People's Hospital of Huaihua City, Huaihua, China

^7^. Department of Pharmacy, Yichun People's hospital, Yichun, Jiangxi Province, China

[^+^] These authors contributed equally to this work.

E-mail: [zhouwenhuyaoji@163.com](mailto:zhouwenhuyaoji@163.com)


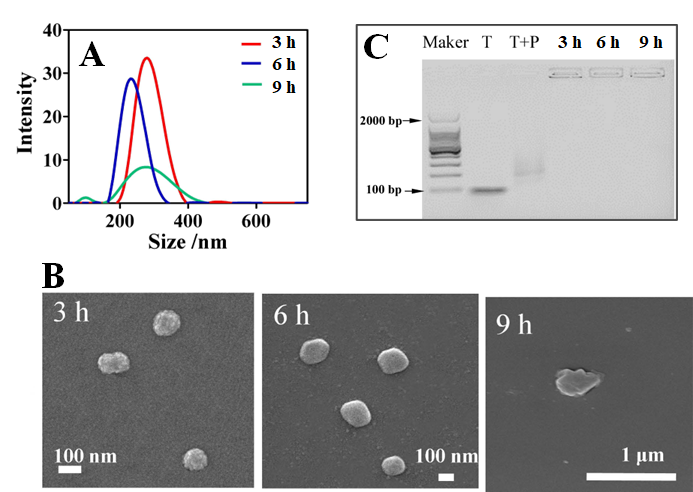


**Figure S1.** Dynamic monitoring the DF formation via RCA by (A) hydrodynamic size, (B) TEM micro-images, and (C) gel electrophoresis.

**Figure S2.** Dynamic monitoring the particle size of CH/DF in PBS buffer and FBS-containing medium over 24 h.


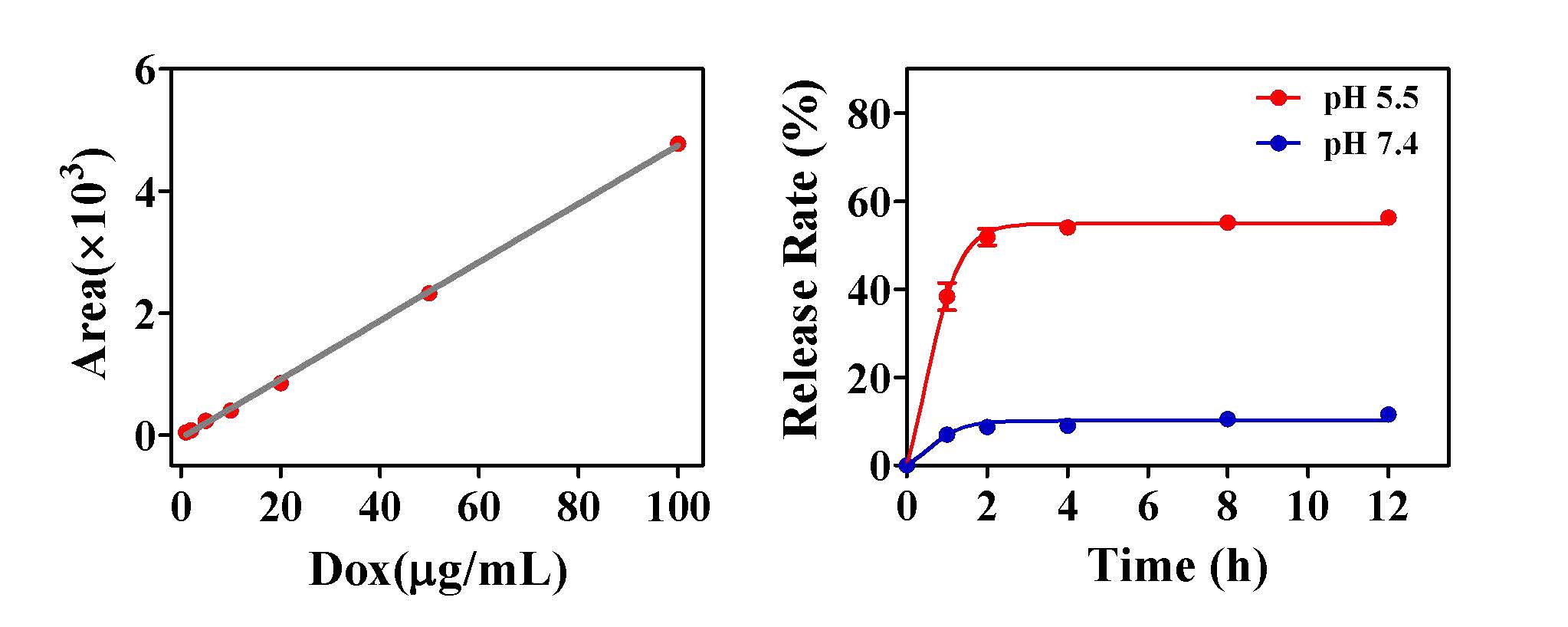


**Figure S3.** Kinetics of drug release in different buffer conditions.

**Figure S4.** Multiple turn-over test of the nanozyme. The reaction was performed for 80 s, and then the nanoparticles were collected by centrifugation. Next, the nanoparticles were re-dispersed for next round of reaction.

**Figure S5.** ^1^O_2_ generation upon laser irradiation for CH/DF and CH/G4 without addition of H_2_O_2_.

**Figure S6.** ^1^O_2_ generation upon laser irradiation for CH/DF and CH/G4 in solution.

**Figure S7.** Dynamic monitoring the body weight of the mice during treatments.


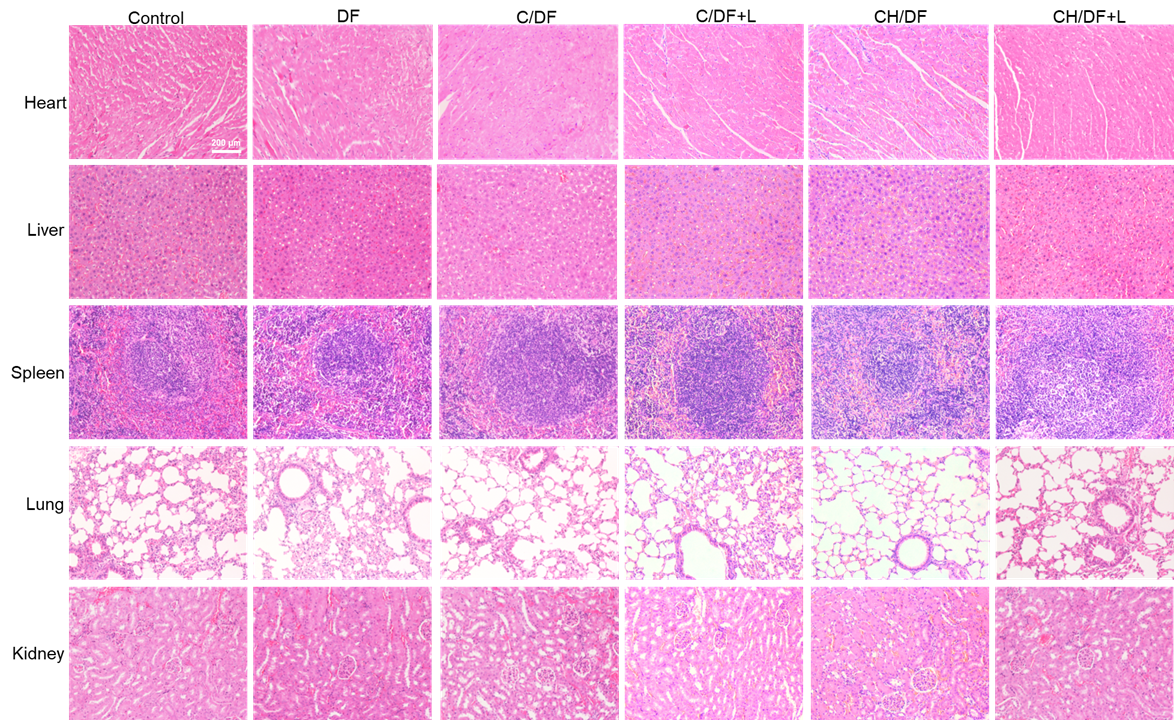


**Figure S8.** H&E staining the major organs after various treatments.
